# miR-30a-3p Regulates Autophagy in the Involution of Mice Mammary Glands

**DOI:** 10.3390/ijms241814352

**Published:** 2023-09-20

**Authors:** Lei Tian, Shancheng Guo, Zhiye Zhao, Yuxu Chen, Chunmei Wang, Qingzhang Li, Ye Li

**Affiliations:** 1 Faculty of Food Science and Engineering, Kunming University of Science and Technology, Kunming 650500, China; leotian@kust.edu.cn (L.T.); at18182832099@163.com (S.G.); a1791366295@163.com (Z.Z.); a1907180236@163.com (Y.C.); 2 Key Laboratory of Dairy Science of Education Ministry, College of Veterinary Medicine, Northeast Agricultural University, Harbin 150030, China; wangcm-1@sohu.com; 3 School of Medicine, Kunming University of Science and Technology, Kunming 650500, China

**Keywords:** autophagy, Atg12, involution, lactation cycle, miRNA, mammary glands

## Abstract

The mammary gland undergoes intensive remodeling during the lactation cycle, and the involution process of mammary gland contains extensive epithelial cells involved in the process of autophagy. Our studies of mice mammary glands suggest that miR-30a-3p expression was low during involution compared with its high expression in the mammary glands of lactating mice. Then, we revealed that miR-30a-3p negatively regulated autophagy by autophagy related 12 (*Atg12*) in mouse mammary gland epithelial cells (MMECs). Restoring ATG12, knocking down autophagy related 5 (*Atg5*), starvation, and Rapamycin were used to further confirm this conclusion. Overexpression of miR-30a-3p inhibited autophagy and altered mammary structure in the involution of the mammary glands of mice, which was indicative of alteration in mammary remodeling. Taken together, these results elucidated the molecular mechanisms of miR-30a-3p as a key induction mediator of autophagy by targeting *Atg12* within the transition period between lactation and involution in mammary glands.

## 1. Introduction

The evolutionary origin of the mammary gland dates to the Carboniferous period nearly 310 million years ago [1]. The mammary glands of mammals are highly conserved in terms of structure, development, and nutritional composition, and the postnatal stage of the mammary gland development cycle involves pregnancy, lactation, and involution [2]. The weaning of offspring regulates the development of the mammary gland from lactation to involution, which leads to remodeling processes and the regression of mammary tissue. Cessation of suckling and milk accumulation triggers this transformation [2]. The involution process of the mammary gland includes extensive epithelial cell death, extracellular matrix remodeling, and recruitment of immune cells [3]. These events are regulated by various factors such as hormones, cytokines, noncoding RNAs, etc. Mammary involution of rodents consists of two main phases: the reversible phase (0–2 days) and the irreversible phase (8–18 days) [4]. Zinc transporter 2 regulates lysosomal-mediated cell death during lactation to a reversible phase of involution [5]. Estrogen promotes involution by exacerbating inflammation, cell death, and adipocytes’ repopulation in BALB/c mice [6].

Autophagy and apoptosis play important roles in cell death and removal during the mammary involution of mice, and autophagy mediates efferocytosis and suppresses inflammation in this process [7]. However, the specific roles of autophagy in cell survival or death in mammary involution have remained debatable [8]. Autophagy plays a key role in the transition from reversible to irreversible involution and regulates mammary gland involution by restraining apoptosis-driven irreversible changes [9]. Cows exposed to heat stress had extended involution due to delayed autophagy signaling, which impaired overall mammary gland turnover during the dry period [10]. Signal transducer and activator of transcription 3 (STAT3) target genes p55α/p50α regulated autophagy and cell death during the mammary involution of mice [11].

The expression of microRNAs (miRNAs) has time- and tissue-specific characteristics, and miRNAs are differentially expressed in the mammary gland at different developmental stages [12,13]. The miR-145 level increased in the lactating mammary gland of dairy cows, and miR-145 regulated fatty acid metabolism by targeting forkhead box protein O1 to affect sterol regulatory element-binding protein 1 activity in bovine mammary epithelial cells [14]. Our previous works had shown that let-7g-5p decreased in the mammary glands of mice during pregnancy and regulated mammary cells’ differentiation and function by targeting protein kinase C alpha [15]. In addition, we showed that miR-142-3p decreased in the mammary glands of lactating mice and regulated milk synthesis and the structure of prolactin receptor-mediated multiple signaling pathways [16].

In mice mammary glands, miR-30a-3p was downregulated during involution compared with its level during lactation. We revealed that miR-30a-3p negatively regulated autophagy by *Atg12* in MMECs. Restoring ATG12, knocking down *Atg5*, starvation, and rapamycin were used to further confirm this conclusion. Overexpression of miR-30a-3p inhibited autophagy and altered mammary structure in the mammary involution of mice, which was indicative of alteration in mammary remodeling. These results elucidated the molecular mechanisms of miR-30a-3p’s regulation of mammary involution.

## 2. Results

### 2.1. miR-30a-3p Was Downregulated during Involution Compared with Lactation in Mice Mammary Glands

**Figure 1:**
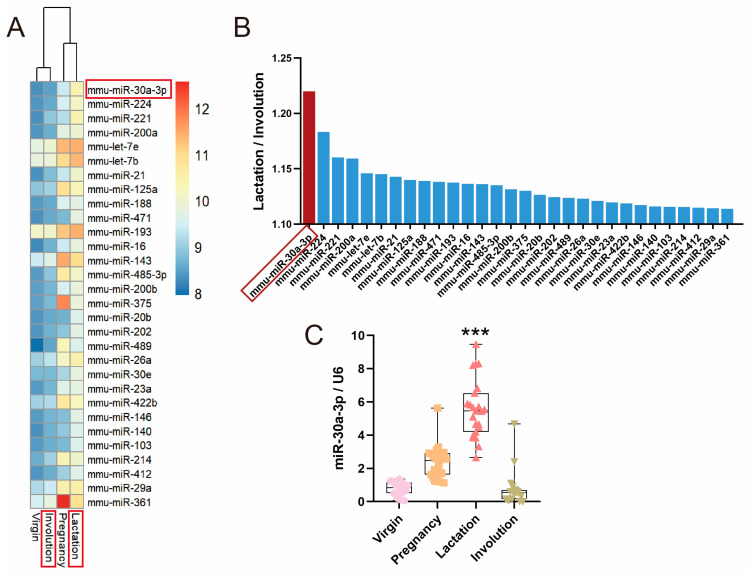
miR-30a-3p was downregulated during involution compared with lactation in mice mammary glands. (**A**) Heat map illustrating the miRNAs’ expression in mouse mammary gland of four developmental stages, picking up miRNAs with the top 30 ratio of lactation/involution. (**B**) miRNAs with the top 30 ratio of lactation/involution. (**C**) The expression of miR-30a-3p in mouse mammary gland of four developmental stages. Data are presented as mean ± SD, *** *p* < 0.001.

In previous research, miRNA microarray was used to compare the miRNA expression profiles in mouse mammary glands of four developmental stages [12]: virgin, pregnancy, lactation, and involution (Figure 1A). We found that miR-30a-3p was most significantly downregulated during involution compared with during lactation (Figure 1B). The result of quantitative real-time PCR (qRT-PCR) showed that expression of miR-30a-3p was also decreased in the involution of the mammary glands of mice (Figure 1C; *p* < 0.001).

### 2.2. miR-30a-3p Negatively Regulated Autophagy in MMECs

**Figure 2:**
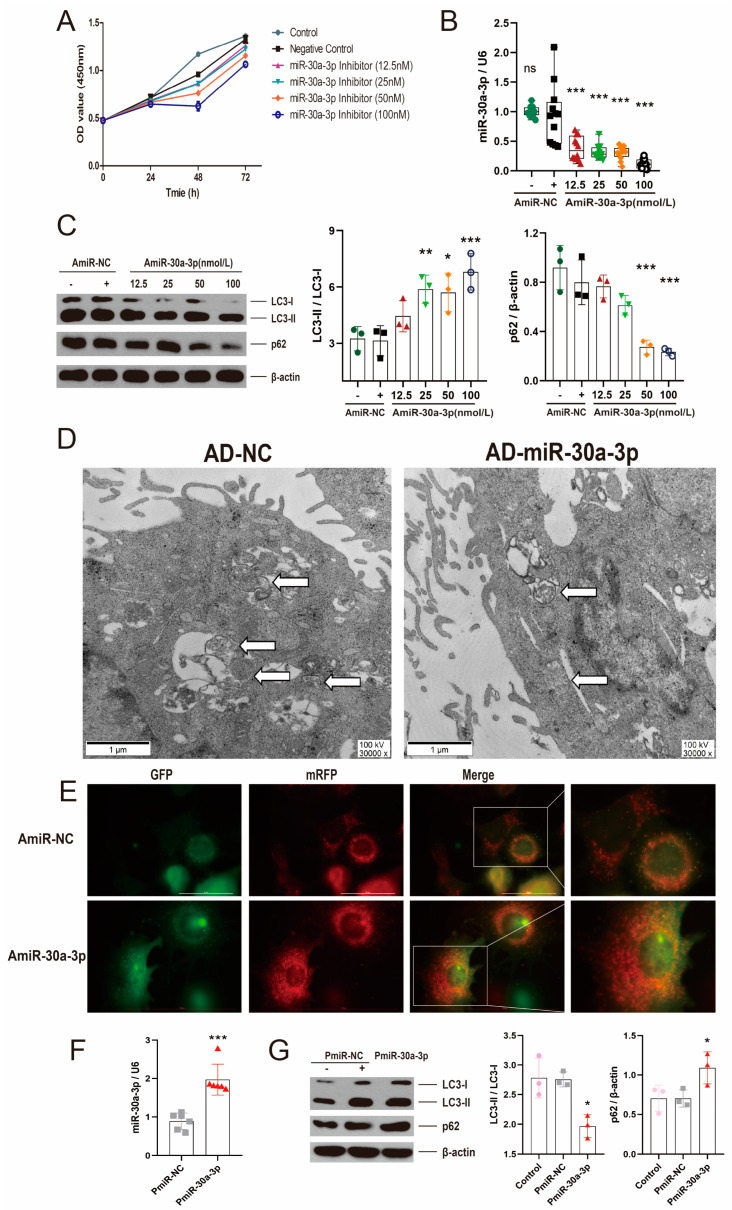
miR-30a-3p negatively regulated autophagy in MMECs. (**A**) The effect of several concentrations of miR-30a-3p inhibitor on MMECs’ viability at different times. (**B**) miR-30a-3p levels for MMECs of various concentrations of inhibitor treatment (AmiR-30a-3p) and MMECs of control treatment (AmiR-NC), as measured by qRT-PCR. (**C**) LC3-II/LC3-I and p62 levels of MMECs for AmiR-NC and AmiR-30a-3p, as measured by Western blot. (**D**) TEM images for MMECs overexpressing miR-30a-3p (AD-miR-30a-3p) and negative control (AD-NC). White arrows indicate autophagosomes. (**E**) Representative immunofluorescence staining of MMECs’ autophagosomes for AmiR-NC and AmiR-30a-3p. Scale bars, 50 μm. (**F**) miR-30a-3p levels for MMECs of pEGFP-miR30a-3p treatment (PmiR-30a-3p) and MMECs of control treatment (PmiR-NC), as measured by qRT-PCR. (**G**) LC3-II/LC3-I and p62 levels of MMECs for PmiR-NC and PmiR-30a-3p, as measured by Western blot. LC3: Microtubule-associated protein light chain 3. Data are presented as mean ± SD, * *p* < 0.05, ** *p* < 0.01, *** *p* < 0.001.

To investigate the effect of miR-30a-3p inhibitor in MMECs, the cell viabilities treated by several concentrations inhibitors were determined at different times via Cell Counting Kit 8 (CCK8). miR-30a-3p inhibitor (100 nM) decreased (*p* < 0.01) cell viability at 48 h. (Figure 2A). The MMECs were harvested after transfection with several concentrations’ inhibitor of miR-30a-3p at 48 h. The result of qRT-PCR revealed that miR-30a-3p was significantly reduced by various concentrations’ inhibitor (AmiR-30a-3p) (Figure 2B; all, *p* < 0.001). To further confirm miR-30a-3p’s function in autophagy, we examined microtubule-associated protein light chain 3 II (LC3-II), LC3-I, and p62 levels after MMECs were treated with miR-30a-3p inhibitor. Western blot analysis showed that LC3-II/LC3-I were increased with 12.5 nM, 50 nM, and 100 nM of inhibitor (Figure 2C; *p* < 0.01, *p* < 0.05, *p* < 0.001), and p62 was decreased with 50 nM and 100 nM of inhibitor (Figure 2C; all, *p* < 0.001).

In addition, transmission electron microscopy (TEM) results showed that autophagosomes were decreased when miR-30a-3p (AD-miR-30a-3p) was overexpressed in MMECs (Figure 2D). Conversely, immunofluorescent staining revealed that autophagosomes were notably increased by miR-30a-3p inhibitor (50 nM) (Figure 2E). Vector pEGFP-miR30a-3p was constructed by the stable expression of miR-30a-3p (Figure S1C), and pEGFP-miR30a-3p was verified by restriction enzyme (Figure S1A) and sequencing (Figure S1B). In MMECs, miR-30a-3p was significantly upregulated (Figure 2F; *p* < 0.001), LC3-II/LC3-I was decreased, and p62 was increased (Figure 2G; all, *p* < 0.05) by pEGFP-miR30a-3p (PmiR-30a-3p). Thus, these data demonstrated that miR-30a-3p negatively regulated autophagy in MMECs.

### 2.3. miR-30a-3p-Regulated Autophagy Verified by Bafilomycin A1 in MMECs

**Figure 3:**
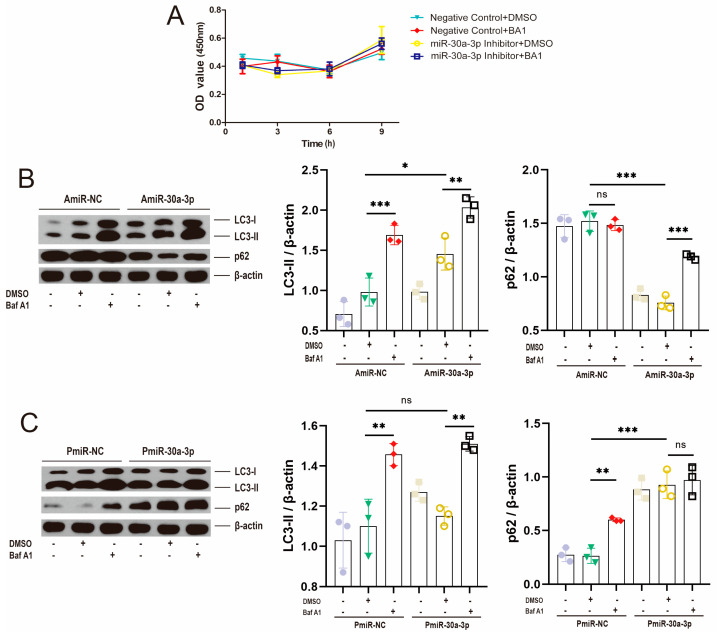
Baf A1 restored the miR-30a-3p regulation on autophagy in MMECs. (**A**) The effect of Baf A1 when miR-30a-3p decreased expression of MMECs’ viability at different times. (**B**) LC3-II and p62 levels of MMECs for Baf A1 and/or AmiR-30a-3p, as measured by Western blot. (**C**) LC3-II and p62 levels of MMECs for Baf A1 and/or PmiR-30a-3p, as measured by Western blot. LC3: Microtubule-associated protein light chain 3. Data are presented as mean ± SD, ns: no significance, * *p* < 0.05, ** *p* < 0.01, *** *p* < 0.001.

Bafilomycin A1 (Baf A1) hinders the autophagy process by blocking fusion between autophagosomes and lysosomes as well as lysosomal degradation [17]. Cell viability assay was performed at 1–9 h after incubation with Baf A1, miR-30a-3p inhibitor (AmiR-30a-3p), and their negative control (AmiR-NC), and the results showed that, for each group, there was no significant effect on the cell viability of MMECs (Figure 3A; *p* > 0.05). MMECs were incubated with miR-30a-3p inhibitor and cotreated with DMSO and/or Baf A1 for 48 h. Western blot analysis revealed that miR-30a-3p inhibitor increased LC3-II (*p* < 0.05) and decreased p62 (*p* < 0.001), indicating the development of autophagy, and Baf A1 upregulated LC3-II in AmiR-NC and AmiR-30a-3p (*p* < 0.001, *p* < 0.01). However, Baf A1 did not affect p62 (*p* > 0.05) in AmiR-NC, whereas the p62 level was upregulated by Baf A1 in AmiR-30a-3p (*p* < 0.001) (Figure 3B). Moreover, pEGFP-miR30a-3p did not significantly effect LC3-II (*p* > 0.05), but increased p62 (*p* < 0.001), suggesting the inhibition of autophagy. Baf A1 both upregulated LC3-II levels in PmiR-NC and PmiR-30a-3p (all, *p* < 0.01), and p62 level was upregulated by Baf A1 in PmiR-NC (*p* < 0.01). However, Baf A1 did not affect p62 (*p* > 0.05) in PmiR-30a-3p (*p* > 0.05) (Figure 3C). Taken together, these data verified that miR-30a-3p regulated autophagy in MMECs by Baf A1.

### 2.4. miR-30a-3p Might Inhibit Autophagy in MMECs by Atg12

**Figure 4:**
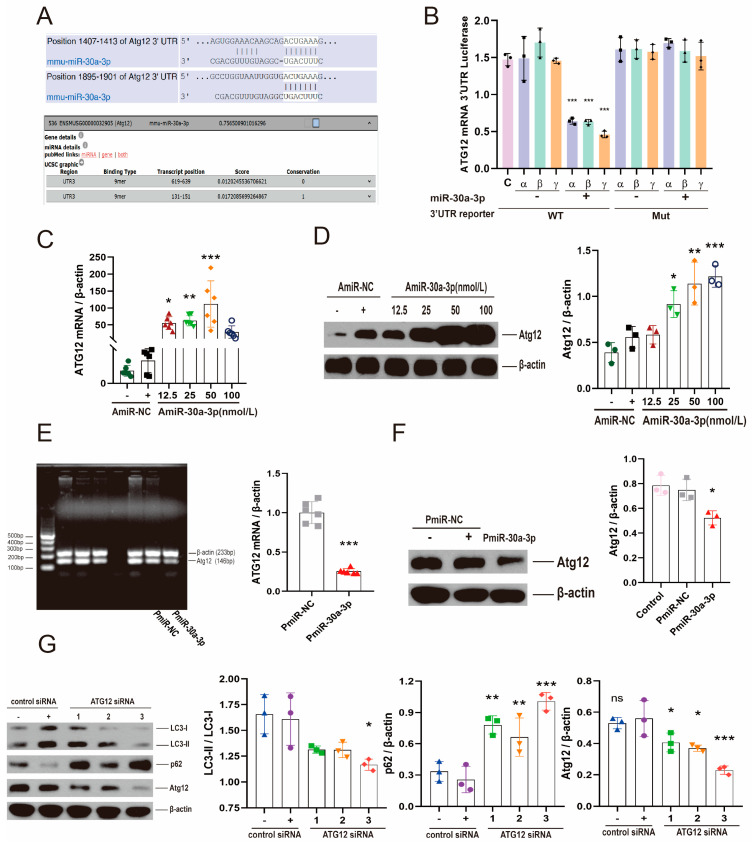
miR-30a-3p regulated autophagy by targeting *Atg12*. (**A**) *Atg12* was identified as a predicted target of miR-30a-3p. (**B**) The luciferase assay of miR-30a-3p and *Atg12* (**C**) *Atg12* mRNA levels for MMECs of various concentrations of inhibitor treatment (AmiR-30a-3p) and MMECs of control treatment (AmiR-NC), as measured by qRT-PCR. (**D**) ATG12 expression of MMECs for AmiR-NC and AmiR-30a-3p, as measured by Western blot. (**E**) *Atg12* mRNA levels for MMECs of pEGFP-miR30a-3p treatment (PmiR-30a-3p) and control treatment (PmiR-NC), as measured by qRT-PCR. (**F**) ATG12 expression of MMECs for PmiR-NC and PmiR-30a-3p, as measured by Western blot. (**G**) LC3-II/LC3-I, p62, and ATG12 levels for MMECs of *Atg12* siRNA treatment and MMECs of control siRNA treatment, as measured by Western blot. ATG12: autophagy related 12; LC3: microtubule-associated protein light chain 3. Data are presented as mean ± SD, ns: no significance, * *p* < 0.05, ** *p* < 0.01, *** *p* < 0.001.

Atg12 was identified as a predicted target of miR-30a-3p by TargetScanMouse 7.1 (http://www.targetscan.org/mmu_71/ URL (accessed on January 2016)) and microT-CDS (DIANA TOOLS) (http://diana.imis.athena-innovation.gr/DianaTools/ URL (accessed on July 2012)), and potential binding target sites of miR-30a-3p were 1407 and 1895 in a 3′-untranslated region (3′-UTR) of Atg12 mRNAs (Figure 4A). To verify the target sites of miR-30a-3p in Atg12 mRNA 3′-UTR, we cloned the 3′-UTR 1407 (α), 1895 (β), and 1407 + 1895 (γ) sites into pMIR-REPORT™ Luciferase vector and co-transfected these vectors with miR-30a-3p mimic or the scrambled negative control into 293T cells, respectively. A β-gal control vector was used as a reference control. Mutants with the putative binding sites (Mut) were prepared as described in Materials and Methods. Luciferase activity of 293T cells transfected with miR-30a-3p mimic and wild-type (WT) binding sites were significant decreased (all, *p* < 0.001) compared with negative control and WT vector, negative control and Mut vector, and miR-30a-3p mimic with Mut vector (Figure 4B).

The result of qRT-PCR showed that the Atg12 mRNAs were dramatically increased by 12.5 nmol/L, 25 nmol/L, and 50 nmol/L inhibitor (Figure 4C; *p* < 0.05, *p* < 0.01, *p* < 0.001). Similar results were validated by Western blot analysis of ATG12 (Figure 4D). To further confirm the relationship between miR-30a-3p and ATG12, we examined Atg12 mRNA and Atg12 protein after MMECs were treated with pEGFP-miR30a-3p. The results indicated that Atg12 mRNA (Figure 4E; *p* < 0.001) and ATG12 protein (Figure 4F; *p* < 0.05) were downregulated by pEGFP-miR30a-3p.

Next, we knocked down ATG12 by Atg12 siRNA, and we found that ATG12 and LC3-II/LC3-I were significantly decreased (Figure 4G; *p* < 0.001, *p* < 0.05); conversely, p62 was increased (*p* < 0.001) by *Atg12* siRNA-3. In summary, these data indicated that ATG12 was a target of miR-30a-3p, and suggested that miR-30a-3p regulated autophagy in MMECs by ATG12.

### 2.5. Restoring ATG12 and Knocking down ATG5 Were Used to Validate That miR-30a-3p Inhibited Autophagy in MMECs by Atg12

**Figure 5:**
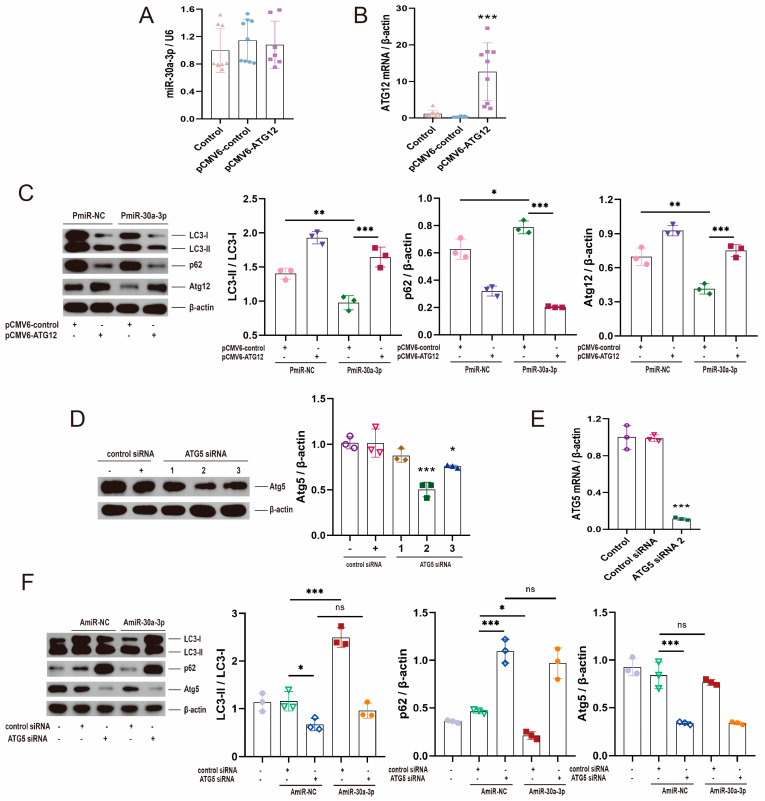
Restoring ATG12 and knocking down ATG5 were used to validate that miR-30a-3p inhibited autophagy in MMECs by ATG12. (**A**) miR-30a-3p levels for MMECs of pCMV6-*Atg12* treatment and MMECs of pCMV6-control treatment, as measured by qRT-PCR. (**B**) *Atg12* mRNA levels for MMECs of pCMV6-*Atg12* treatment and MMECs of pCMV6-control treatment, as measured by qRT-PCR. (**C**) LC3-II/LC3-I, p62, and ATG12 levels of MMECs for pCMV6-*Atg12* and/or PmiR-30a-3p, as measured by Western blot. (**D**) ATG5 expression for MMECs of *Atg5* siRNA treatment and MMECs of control siRNA treatment, as measured by Western blot. (**E**) *Atg5* mRNA levels for MMECs of *Atg5* siRNA treatment and MMECs of control siRNA treatment, as measured by qRT-PCR. (**F**) LC3-II/LC3-I, p62, and ATG5 levels for *Atg5* siRNA and/or AmiR-30a-3p, as measured by Western blot. ATG5: autophagy related 5; ATG12: autophagy related 12; LC3: microtubule-associated protein light chain 3. Data are presented as mean ± SD, ns: no significance, * *p* < 0.05, ** *p* < 0.01, *** *p* < 0.001.

The pCMV6-Atg12 vector did not exert a significant effect on miR-30a-3p levels (Figure 5A; *p* > 0.05) but upregulated ATG12 expression (Figure 5B; *p* < 0.001). To validate the effect of miR-30a-3p on autophagy in MMECs by ATG12, we upregulated miR-30a-3p level by pEGFP-miR30a-3p (PmiR-30a-3p), then increased ATG12 expression by pCMV6-Atg12. Western blot revealed that overexpression of miR-30a-3p decreased LC3-II/LC3-I (*p* < 0.01) and ATG12 (*p* < 0.01), and increased p62 (*p* < 0.05), but these tendencies were reversed by restoring ATG12 (Figure 5C).

Next, we knocked down ATG5 by Atg5 siRNA, and Western blot analysis showed that Atg5 level was significantly downregulated by Atg5 siRNA-2 (Figure 5D; *p* < 0.001); the same result was verified by qRT-PCR (Figure 5E; *p* < 0.001). After that, we downregulated the miR-30a-3p level by inhibitor (AmiR-30a-3p), and we decreased ATG5 expression by Atg5 siRNA-2. Western blot revealed that suppression of miR-30a-3p increased LC3-II/LC3-I (*p* < 0.001), decreased p62 (*p* < 0.05), and did not exert a significant effect on ATG5 (*p* > 0.05), but these tendencies were smoothed (*p* > 0.05) by knocking down ATG5 (Figure 5F). Overall, these results confirmed that miR-30a-3p inhibited autophagy in MMECs by ATG12.

### 2.6. miR-30a-3p’s Regulation of Autophagy by ATG12 Was Verified through Starvation and Rapamycin In Vitro

**Figure 6:**
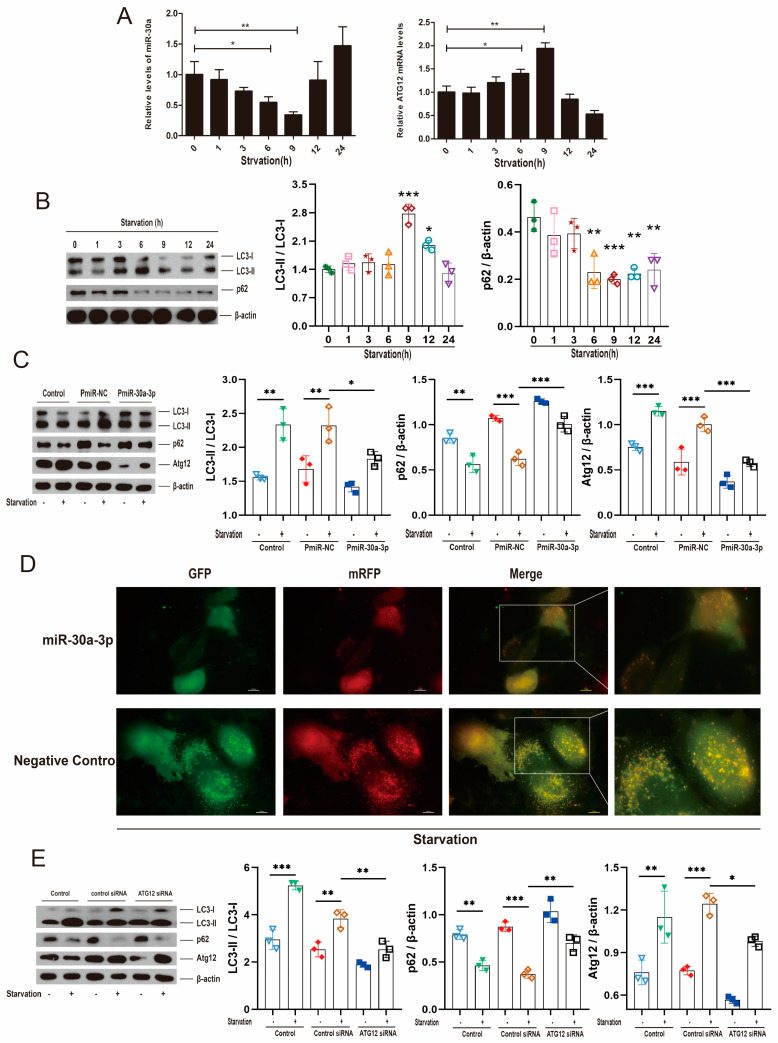
miR-30a-3p regulated autophagy by ATG12; this was verified through starvation. (**A**) miR-30a-3p and *Atg12* mRNA levels in MMECs at different starvation times, as measured by qRT-PCR. (**B**) LC3-II/LC3-I and p62 levels in MMECs at different starvation times, as measured by Western blot. (**C**) LC3-II/LC3-I, p62 and ATG12 levels of MMECs for starvation and/or PmiR-30a-3p, as measured by Western blot. (**D**) Representative immunofluorescence staining of MMECs’ autophagosomes after 9 h of starvation and/or overexpression of miR-30a-3p. Scale bars, 10 μm. (**E**) LC3-II/LC3-I, p62, and ATG12 levels of MMECs for starvation and/or *Atg12* siRNA, as measured by Western blot. ATG12: autophagy related 12; LC3: microtubule-associated protein light chain 3. Data are presented as mean ± SD, * *p* < 0.05, ** *p* < 0.01, *** *p* < 0.001.

To investigate the effect of starvation (0.5% fetal bovine serum, FBS) on the autophagy of MMECs, Atg12 mRNA and miR-30a-3p levels were determined at different starvation times via qRT-PCR. The results showed that miR-30a-3p levels were decreased (*p* < 0.05, *p* < 0.01) at 6 h and 9 h; in contrast, Atg12 mRNA levels were increased (*p* < 0.05, *p* < 0.01) at 6 h and 9 h (Figure 6A). Western blot analysis revealed that LC3-II/LC3-I were increased (*p* < 0.001, *p* < 0.05) by starvation at 9 h and 12 h, and p62 was decreased by starvation at 6–24 h (Figure 6B). Then, we starved cells for 9 h and upregulated miR-30a-3p level by pEGFP-miR30a-3p (PmiR-30a-3p). Western blot analysis showed that starvation increased LC3-II/LC3-I (all, *p* < 0.01) and ATG12 (all, *p* < 0.001) decreased p62 (*p* < 0.01, *p* < 0.001), but these tendencies were reversed by overexpression of miR-30a-3p (Figure 6C). Similarly, immunofluorescent staining revealed that autophagosomes were notably increased by starvation at 9 h, but the increasing trends were smoothed by overexpression of miR-30a-3p (Figure 6D). Additionally, we starved cells for 9 h and downregulated the ATG12 level by Atg12 siRNA. Western blot showed that starvation increased LC3-II/LC3-I (*p* < 0.001, *p* < 0.01) and ATG12 (*p* < 0.01, *p* < 0.001) and decreased p62 (*p* < 0.01, *p* < 0.001). However, these tendencies were reversed by knocking down ATG12 (Figure 6E).

**Figure 7:**
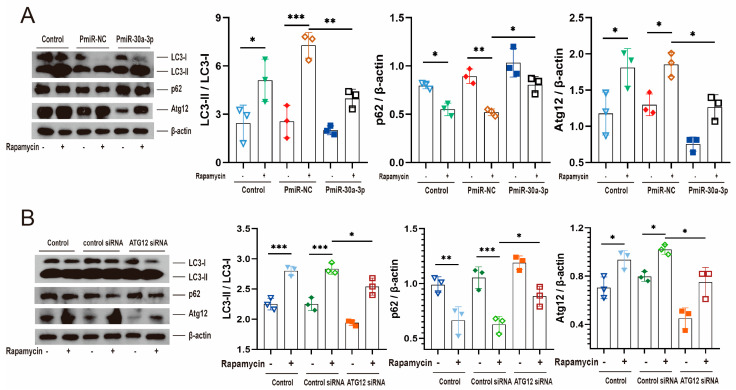
miR-30a-3p regulated autophagy by ATG12. This was verified through rapamycin. (**A**) LC3-II/LC3-I, p62, and ATG12 levels of MMECs for rapamycin and/or PmiR-30a-3p, as measured by Western blot. (**B**) LC3-II/LC3-I, p62, and Atg12 levels of MMECs for rapamycin and/or Atg12 siRNA, as measured by Western blot. ATG12: autophagy related 12; LC3: microtubule-associated protein light chain 3. Data are presented as mean ± SD, * *p* < 0.05, ** *p* < 0.01, *** *p* < 0.001.

Rapamycin induces autophagy by inhibiting the mammalian target of rapamycin, which is the negative regulator of autophagy. To further confirm that miR-30a-3p regulated autophagy in MMECs by ATG12, we treated MMECs with rapamycin and upregulated miR-30a-3p level by pEGFP-miR30a-3p (PmiR-30a-3p). Western blot analysis revealed that rapamycin treatment increased LC3-II/LC3-I (*p* < 0.05, *p* < 0.001) and ATG12 (all, *p* < 0.05) and decreased p62 (*p* < 0.05, *p* < 0.01). These tendencies were reversed by the overexpression of miR-30a-3p (Figure 7A). Similar experiments were performed by rapamycin treatment and knocking down ATG12, and similar results were observed (Figure 7B). Taken together, we confirmed that miR-30a-3p inhibited autophagy in MMECs by Atg12.

### 2.7. Overexpression of miR-30a-3p Inhibited Autophagy and Delayed Mice Mammary Involution

**Figure 8:**
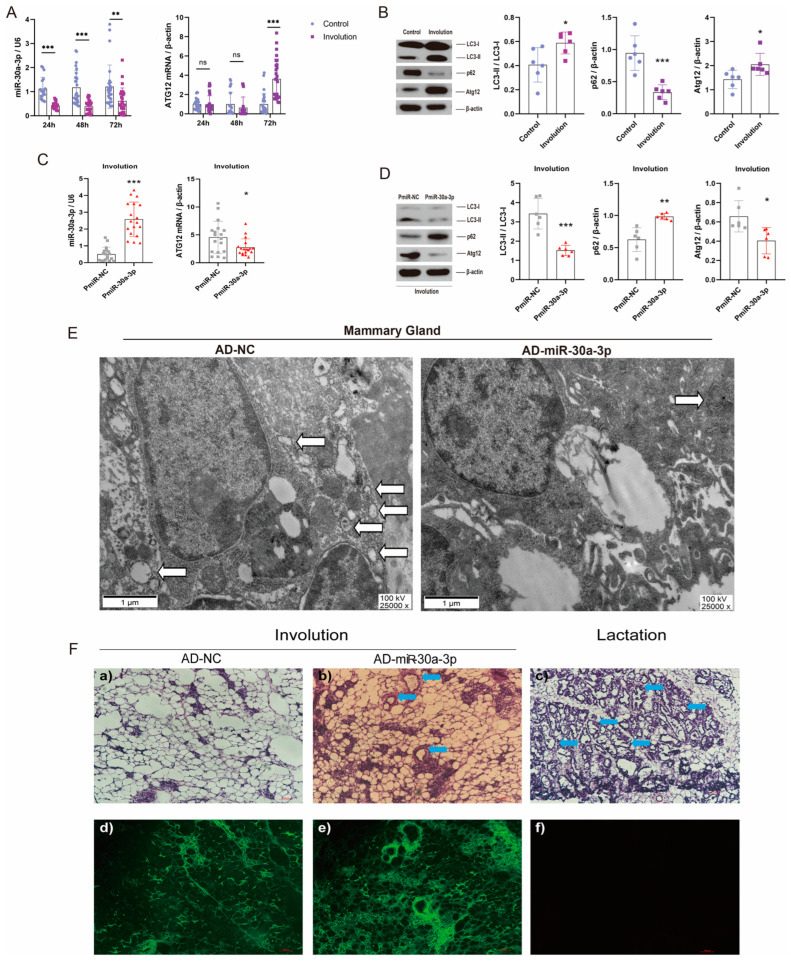
Overexpression of miR-30a-3p inhibited autophagy and delayed mice mammary involution. (**A**) miR-30a-3p and *Atg12* mRNA levels in mice mammary glands at different forced weaning times, as measured by qRT-PCR. (**B**) LC3-II/LC3-I, p62, and ATG12 levels in involuting mammary glands, as measured by Western blot. (**C**) miR-30a-3p and *Atg12* mRNAs levels in involuting mammary glands after pEGFP-miR30a-3p treatment (PmiR-30a-3p) and control treatment (PmiR-NC), as measured by qRT-PCR. (**D**) LC3-II/LC3-I, p62, and ATG12 levels in involution mammary glands following pEGFP-miR30a-3p treatment (PmiR-30a-3p) and control treatment (PmiR-NC), as measured by Western blot. (**E**) TEM images of involuting mammary glands showing overexpression of miR-30a-3p (AD-miR-30a-3p) and negative control (AD-NC). White arrows indicate autophagosomes. (**F**) Mammary acini and ducts (blue arrows) were increased by overexpression of miR-30a-3p (AD-miR-30a-3p) in involuting mammary glands, which delayed the involution. ATG12: autophagy related 12; LC3: microtubule-associated protein light chain 3. Data are presented as mean ± SD, * *p* < 0.05, ** *p* < 0.01, *** *p* < 0.001.

Lactating mice were forced to wean, which induced the mammary glands from lactation to involution [18]. miR-30a-3p and Atg12 mRNA levels were determined at several times after forced weaning. At 24 h, 48 h, and 72 h after forced weaning, miR-30a-3p levels were all downregulated (*p* < 0.001, *p* < 0.001, *p* < 0.01), but Atg12 mRNA levels were upregulated (*p* < 0.001) at 72 h (Figure 8A). Next, Western blot analysis revealed that LC3-II/LC3-I and ATG12 were increased (all, *p* < 0.05) and p62 was decreased (*p* < 0.001) at 72 h after forced weaning (involution) (Figure 8B). To further investigate the effect of miR-30a-3p on autophagy in the involution of mammary glands, we upregulated miR-30a-3p levels by pEGFP-miR30a-3p in involuting mammary glands. qRT-PCR data showed that miR-30a-3p levels were increased (*p* < 0.001), and Atg12 mRNA levels were decreased (*p* < 0.05) by pEGFP-miR30a-3p in involuting mammary glands (Figure 8C). Western blot analysis revealed that overexpression of miR-30a-3p decreased LC3-II/LC3-I (*p* < 0.001) and Atg12 (*p* < 0.05) but increased p62 (*p* < 0.01) in involuting mammary glands (Figure 8D). Further, TEM results showed that autophagosomes were decreased when miR-30a-3p (AD-miR-30a-3p) was overexpressed in involuting mammary glands (Figure 8E). Conspicuously, mammary acini and ducts were increased by the overexpression of miR-30a-3p in involuting mammary glands (Figure 8F).

## 3. Discussion

Postpartum mammary involution is a normal process and is accompanied by secretory epithelial cell death, alveolar regression, gland repopulation by adipocytes, and stroma remodeling [19,20]. This process has received wide attention, since mammary involution has been involved in pregnancy-associated breast cancer and is associated witgh higher metastatic probability [21,22]. A series of cell death modes have been identified in mammary involution, such as apoptosis, lysosomal permeabilization, and autophagy, but the mechanisms and significance of the different death modes are unclear [23]. The level of estrogen receptor α is increased in virgin cows and decreased in lactating and involuting cows [24]. Prolactin expression in rats is upregulated in the middle to late stages of pregnancy and then drops during the early stages of involution [25]. STAT3 is activated by leukemia inhibitory factor in bovine mammary involution [26]. Mammary gland protein-40 is catalytically inactivated during buffalo mammary gland involution [27]. In summary, mammary involution is regulated by various signaling molecules. To further explore the mechanisms of involution, miRNA microarray was used to compare the miRNA expression profiles of mouse mammary gland in four developmental stages. We found that miR-30a-3p was most significantly downregulated during involution compared with lactation.

miRNAs are involved in the regulation of many biological processes, for example, cell proliferation, differentiation, apoptosis, and migration, by binding to specific sites of targets [28]. Previous studies demonstrated that miR-30a-3p expression exerts tumor-suppressive functions in some forms of cancers [29,30,31], and miR-30a-3p represses growth, migration, and inflammatory response in rheumatoid arthritis fibroblastic synovial cells [32]. We found that miR-30a-3p negatively regulated autophagy in MMECs and verified this conclusion by Baf A1 (autophagy inhibitor).

To further investigate the specific mechanism, TargetScanMouse 7.1 and microT-CDS (DIANA TOOLS) databases were used to predict the target of miR-30a-3p. We discovered that the potential binding target sites of miR-30a-3p were 3′-UTR of *Atg12* mRNAs, which was consistent with the findings of some studies in the literature [31,33]. The predicted binding sites of miR-30a-3p were 1407 and 1895 in ATG12 3′-UTR (NM_026217). We cloned the 1407, 1895, and 1407 + 1895 sites into pMIR-REPORT™ Luciferase vector to verify the effect of miR-30a-3p on *Atg12*. The results suggested that miR-30a-3p bound both 1407 and 1895 with *Atg12* 3′-UTR. ATG12 is an autophagy-related protein that contributes to proteasome degradation in the autophagy process [34]. The conjugation of the ubiquitin-like protein ATG12 to a lysine residue of ATG5 associates noncovalently with ATG16, which is essential for autophagosome formation [35]. Thus, restoring ATG12 and knocking down ATG5 were used, in our work, to validate the finding that miR-30a-3p inhibited autophagy in MMECs by ATG12.

Nutrient starvation induced a significant increase in autophagy by Ulk1 dephosphorylation and its subsequent dissociation from protein kinase AMP-activated catalytic subunit alpha 1 [36]. We investigated the effect of starvation on the autophagy of MMECs and found that miR-30a-3p was decreased, *Atg12* mRNA was increased, and autophagy was remarkably induced by starvation. However, upregulated miR-30a-3p and downregulated ATG12 reversed these tendencies. Rapamycin promotes autophagy markedly and has been used as a potent inducer of autophagy [37]. Our works revealed that rapamycin treatment increased ATG12 expression and autophagy, but these tendencies were reversed by the overexpression of miR-30a-3p and by knocking down ATG12. Thus, miR-30a-3p regulated autophagy by ATG12; this was verified through starvation and rapamycin.

After weaning, alteration of mammary cytokine and prolactin levels trigger mammary gland involution [38]. Lactating mice were forced to wean, miR-30a-3p was downregulated, ATG12 was upregulated, and autophagy was promoted in mice mammary glands. To further explore the effect of miR-30a-3p on involuting mammary glands, we upregulated miR-30a-3p levels in mammary involution. Experimental data showed that autophagy was inhibited and mammary acini and ducts were increased by the overexpression of miR-30a-3p.

## 4. Materials and Methods

### 4.1. Mice

BALB/c mice were purchased from Harbin Veterinary Research Institute (Harbin, Heilongjiang, China). Mammary tissues of female mice in different periods (virgin: 4, 5, 7 weeks; pregnant: 5, 13, 18 days; lactating: 3, 7, 13 days; involuting: 2, 5, 10 days) were collected, and six mice were used for each group. The mice were sacrificed, and mammary glands were harvested and frozen in liquid nitrogen for further analysis. All animal experiments were performed in accordance with guidelines and regulations of the Northeast Agricultural University Animal Care and Use Committee (2019-2, Harbin, China) and the Kunming University of Science and Technology Animal Ethics Committee (PZWH(DIAN)K2023-0026).

### 4.2. Cell Culture

The HC11 murine mammary gland epithelial cell line and 293T cell line were purchased from National Collection of Authenticated Cell Cultures (Shanghai, China). HC11 was grown at 5% CO_2_ in 1640 (Gibco, Cheshire, CT, USA), with 10% fetal bovine serum (FBS, Gibco, Cheshire, CT, USA), 1% sodium pyruvate (Gibco, Cheshire, CT, USA), and 1% glutamine (Gibco, Cheshire, CT, USA) at 37 °C. The 293T cell line was grown at 5% CO_2_ in DMEM (Gibco, Cheshire, CT, USA), with 10% FBS (Gibco, Cheshire, CT, USA), 1% sodium pyruvate (Gibco, Cheshire, CT, USA), and 1% glutamine (Gibco, Cheshire, CT, USA) at 37 °C. HC11 cells were transfected with miR-30a-3p inhibitor (5′-3′: CUUUCAGUCGGAUGUUUGCAGC, Genepharma, Shanghai, China), *Atg12* siRNA, *Atg5* siRNA, negative control (Genepharma, Shanghai, China), pEGFP-C1, pEGFP-miR-30a-3p, pCMV6-*Atg12*, and control plasmids using Lipofectamine 3000 (Invitrogen, Carlsbad, CA, USA) according to manufacturer’s protocol. Baf A1 (50 nM; CAS 88899-55-2, InvivoGEN, San Diego, CA, USA) and rapamycin (100 nM, V900930, Sigma-Aldrich, St. Louis, MO, USA) were applied.

### 4.3. Quantitative Real-Time PCR (RT-PCR) Analyses

RNAs were isolated from mammary glands at different time points using Trizol (Invitrogen, Carlsbad, CA, USA). M-MLV Reverse Transcriptase (Thermo Fisher Scientific, Waltham, MA, USA) was used to convert 100 ng RNA to cDNA. SYBR™ Green PCR Master Mix (Thermo Fisher Scientific, Waltham, MA, USA) was used to detect the miR-30a-3p and mRNA expressions, which normalized to the U6 or β-actin and were analyzed using the ^ΔΔ^Ct method.

### 4.4. Cell Viability Assay

The cell proliferation test was based on the cell proliferation reagent CCK8 (DOJINDO LABORATORIES, Kamimashiki-gun, Kumamoto, Japan). After treatment, 10 μL of CCK8 reagent was added to the medium in each well of 96-well plate. The cells were incubated in a humidified atmosphere at 37 °C with 5% CO_2_ in air for 1 h. The plate was shaken thoroughly for 10 s, and absorbance was read at 450 nm. The background absorbance was measured in wells containing only the culture medium and CCK8 solution. Cell proliferation data were obtained from at least three independent experiments with at least three wells in a separate 96-well plate. The mean optical density values corresponding to the untreated controls were taken as 100%. The results were expressed as the percentage of the optical density of treated cells relative to that of untreated controls.

### 4.5. Western Blot Analysis

Proteins were isolated from cells and mammary tissues, as previously described [16]. The primary antibodies used were ATG12 (#4180), ATG5 (#12994), p62 (#5114), and LC3I/II (#4108) from Cell Signaling Tech (Danvers, MA, USA); β-actin (sc-47778) and secondary antibody with HRP from Santa Cruz Biotechnology (Santa Cruz, CA, USA). Bands were visualized with enhanced chemiluminescence reagent (Millipore, Billerica, MA, USA), and β-Actin was the control. Western blotting images were analyzed by Image J (NIH, Bethesda, MD, USA). Western blots are representative of a minimum of three independent experiments.

### 4.6. Vectors

The 3′UTR of *Atg12* (NM_026217) including the predicted sites (α:1407 site, β:1895 site, γ:1407 site-1895 site) for miR-30a-3p seizing was obtained by PCR. Then, the *Atg12* 3′UTR was cloned into the pMIR-REPORT vector (Thermo Fisher Scientific, Waltham, MA, USA). The mutational site was obtained by site-directed mutagenesis. miR-30a-3p or control together with these luciferase vectors were co-transfected into 293T cells by a Lipofectamine 3000 (Invitrogen, Carlsbad, CA, USA) protocol in triplicate. Cells were lysed 48 h after transfection; then, the Dual-Light Luciferase and β-Galactosidase Reporter Gene Assay System (Thermo Fisher Scientific, Waltham, MA, USA) was used to assay the luciferase activity according to the manufacturer’s protocols. Mmu-miR-30a-3p (ENSMUSG00000065405) was obtained by PCR. pEGFP-miR30a-3p vector was constructed with pEGFP-C1 (Promega, Madison, WI, USA) to stabilize expression of miR-30a-3p, and pEGFP-miR30a-3p was verified by restriction enzyme and sequencing.

### 4.7. mRFP-GFP-LC3 Assay

The HC11 cells’ culture medium serum concentration was reduced to 0.5% to leave the cells in a starvation state. Then, we obtained cell samples after 1 h, 3 h, 6 h, 9 h, 12 h, and 24 h of cell starvation and analyzed the autophagic flux. Autophagic flux was measured in cells transfected with the autophagy tandem sensor mRFP-GFP-LC3. mRFP-GFP-LC3 adenovirus (MOI = 50; Hanbio, Shanghai, China) was transfected in 50% volume of the culture media for 2 h. After supplementing with an equal volume of media, cells were transfected for 2 h. Then, the culture media was changed to fresh DMEM for 48 h. Autophagy was observed under fluorescence microscopy (Leica, Am Leitz-Park, Wetzlar, Germany). Autophagosomes were labelled with green fluorescent protein (GFP) (green), and LC3 were labelled with monomeric red fluorescent protein (mRFP) (red). GFP is sensitive to acidity and green fluorescence of GFP decreased when autophagosome were fused with lysosome. The autophagic flux was evaluated by counting the number of GFP and mRFP puncta, and 15 cells were used for quantification in each group.

### 4.8. Immunofluorescence, Hematoxylin, and Eosin Staining

Female mice involuting for 1 day were infected with adenovirus expression vector AD-miR-30a-3p in the fourth mammary tissue and empty vector AD-NC was used as the control. Then, female mice lactating for 21 days were used as the lactation control. After 7 days, the mammary tissues of each group were made frozen sections. The mouse mammary gland tissues fixed in 10% formalin solution were sectioned (4 μM) after dehydration, cleaning, and paraffin embedding. The sections were flattened, pasted, and heated on blank glass slides. Histological evaluations were performed with hematoxylin and eosin (H&E) staining and observed with fluorescence microscopy (Leica, Am Leitz-Park, Wetzlar, Germany).

### 4.9. TEM

The fresh mice mammary gland tissue samples were obtained and quickly fixed, as previously described [15]. Mammary gland tissue samples were fixed with 2.5% glutaraldehyde in 0.1 M PBS (pH 7.2) at 37 °C for 1 h, washed three times in buffer for 10 min, fixed in 1% osmium tetroxide for 1 h at 4 °C, and dehydrated through a graded series of ethanol. The samples were treated with propylene oxide, embedded, and polymerized for 72 h at 70 °C. Sections were cut on an ultramicrotome (Leica, Am Leitz-Park, Wetzlar, Germany) and stained with 5% uranyl acetate for 10 min, followed by 1 min in Sato’s lead stain. TEM was performed on an FEI Tecnai G2 (Hillsboro, OR, USA).

### 4.10. Statistical Analysis

All experiments were performed three times. All statistical analyses used Student’s *t*-tests or one-way analysis of variance (ANOVA, followed by Tukey’s post hoc test). *p* < 0.05 was considered statistically significant. Statistical analyses were performed by GraphPad (Boston, MA, USA) Prism v.8.0.1 software.

## 5. Conclusions

**Figure 9:**
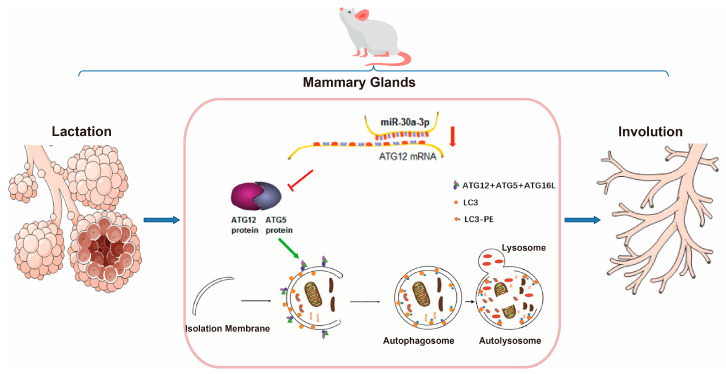
miR-30a-3p regulates autophagy in the involution of mice mammary glands by targeting ATG12. ATG5: autophagy related 5; ATG12: autophagy related 12; ATG16L: autophagy related 16-like 1; LC3: microtubule-associated protein light chain 3.

In conclusion, miR-30a-3p regulated autophagy in involuting mice mammary glands by ATG12, and overexpression miR-30a-3p delayed mammary involution (Figure 9). To our knowledge, our results are the first to clarify the mechanism of miRNA in mammary involution.

## Data Availability

Data will be made available on request.

## Supplementary Materials

The following supporting information can be downloaded at: https://www.mdpi.com/article/10.3390/ijms241814352/s1.

## Abbreviations

3′-UTR: 3′-untranslated region; AD: adenovirus; AmiR-30a-3p: miR-30a-3p inhibitor; AmiR-NC: negative control; ATG: autophagy-related gene; ATG12: autophagy related 12; ATG16L: autophagy related 16-like 1; ATG5: autophagy related 5; Baf A1: Bafilomycin A1; CCK8: Cell Counting Kit 8; EGFP: enhanced green fluorescent protein; FBS: fetal bovine serum; GFP: green fluorescent protein; H&E: hematoxylin and eosin; LC3: microtubule-associated protein light chain 3; miRNAs: microRNAs; MMECs: mouse mammary gland epithelial cells; mRFP: monomeric red fluorescent protein; qRT-PCR: real-time quantitative PCR; Stat3: signal transducer and activator of transcription 3; TEM: transmission electron microscopy; WT: wild-type; β-actin: beta-actin.
