# Supplementary figures and images for: miR-30a-3p Regulates Autophagy in the Involution of Mice Mammary Glands
Source: Int J Mol Sci. 2023 Sep 20;24(18):14352. doi: 10.3390/ijms241814352 (PMC10531886; doi:10.3390/ijms241814352)

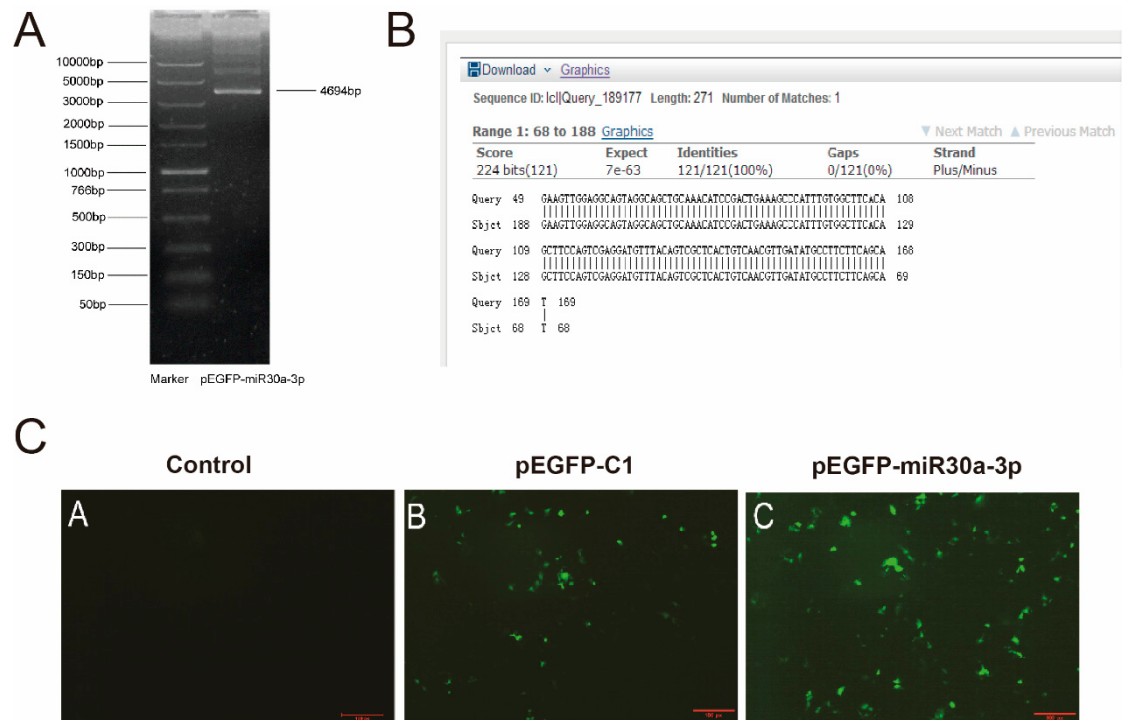

Figure S1 pEGFP-miR30a-3p was verified by restriction enzyme and sequencing.

Supplement: Supplementary file 1 [file ijms-24-14352-s001.zip › ijms-2564176-supplementary.pdf]
